# The Law of “Vis Viva”

**Published:** 1870-03

**Authors:** 


### THE LAW OF "VIS VIVA."

One of the best definitions we have seen of the term *vis viva* is that it is "the measure of mechanical work developed by motive forces or inertia, *in variable motion.*" When the full import of all its terms is comprehended, this definition will be found to accord with the notion of force as precedent to motion. So long as this notion of force prevails, so long must the term *vis viva* or its equivalent be a necessity in the intelligent conception of the laws of motion.

Let us briefly examine this definition with a view to clear away some of the vagueness with which this subject is attended in the popular mind.

What is meant by mechanical work? Certainly, this can be expressed in terms of its accepted unit the foot-pound. A foot-pound is a pound raised one foot without regard to time. This is the *unit of work.* It is not a unit of force, as it is sometimes erroneously considered. More or less force will be required to perform it, according as the time in which it is done is shorter or longer. Power is force in relation to time. The mightiest force requires time to produce an effect. The most infinitesimal force will produce an effect in time.

All this is inseparable from the idea of force as existing independently of matter and antecedent to motion. A mechanical effect is *motion produced;* motion involves the idea of distance traversed ; distance traversed involves the idea of time in which it is traversed. But distance traversed does not necessarily imply mechanical work performed. It is only when a resistance is overcome that work is accomplished. A body moving in absolute space performs no mechanical work, though it move with a constant velocity forever. Let it, however, encounter some other body having less motion and it performs work. It increases the motion of the mass which it strikes against, or some of its particles, or it may produce both these effects. The mass-motion produced is mechanical work. The effect upon the striking body is no less work. Its motion is decreased by the impact.

Increase or decrease of mass motion is, properly speaking, *mechanical work*, and we shall find, upon strict examination, that this is all implied by the term. But as no increase or diminution of motion can take place in a body without its receiving or imparting motion from or to another imparting or receiving body, it follows that *vis viva* practically relates only to transmission of motion from one body to another, in *space and time.*

It will be seen that the idea of *vis viva* is, therefore, essentially different from the term momentum, which is simply the amount of motion a body possesses, considered with relation to definite periods of time and definite distances, and which is expressed by mass or weight multiplied by the time it traverses a definite distance. Momentum has no reference to the amount of motion a body can impart or receive in time and space.

The terms " motive forces " and " inertia " convey the idea of material forces or matter in motion. The expression, " in variable motion," seems unnecessary, since the very idea of imparting or receiving motion implies variable motion. The expression $MV^2$ (mass or weight multiplied into the square of velocity) is the mathematical symbol of *vis viva ;* that is, the measure of the mechanical work developed by a moving body—or in other words the change of motion produced by it on another body in space and time—is measured by its mass multiplied into the square of its velocity.

It must be further observed that *vis viva* is not a measure of force, but of the mechanical work performed, or the change of mass motion produced.

Whether we accept the notion of the existence of occult force which acts upon matter, or accept the doctrine that there is no force which the human mind can recognize other than moving matter, there still remains the necessity for an expression of the law of the transmission of motion. One thing is certain, a body can not transmit motion it does not possess, and if momentum expressed by MV (mass multiplied into velocity) be the absolute amount of motion a body possesses, it certainly can not impart the motion expressed by $MV^2$ or its mass multiplied into the square of its velocity. Evidently there must be some limitation to the interpretation of one or both of these expressions, which will reconcile their apparent conflict. This limitation is, we think, to be found in the fact that in momentum definite spaces and times are considered with uniform motion, while in *vis viva* the motion considered is a variable one, or one in which motion is constantly received or imparted; and that $MV^2$ determines the space through which a body will move before it comes to rest, when opposed by a resistance capable of absorbing all its motion. MV, or momentum, is the expression of the motion of a body neither imparting or receiving motion, and therefore performing no work. Momentum is an absolute expression when the factor of time in the velocity is constant. *Vis viva* is a proportional or relative term only.

Thus a body moving uniformly through a definite space in a definite time, has a momentum expressed by its mass or weight multiplied into its velocity. While passing through the space, or when it has passed over the space, it has the power to overcome a certain constant resistance, and to move a certain distance before it imparts all of its motion to a resisting medium. Its relative or proportional power to move through such a resisting medium or to overcome an attractive force is its *vis viva* ($MV^2$) as compared with other bodies moving through similar media or opposing an equal attractive force. It is not an absolute expression of the quantity of motion in a body, like momentum. It has reference only to space traversed, while motion is being absorbed by resistance.

